# Supplementary material for: Quantifying disorder one atom at a time using an interpretable graph neural network paradigm
Source: Nat Commun. 2023 Jul 7;14:4030. doi: 10.1038/s41467-023-39755-0 (PMC10328988; doi:10.1038/s41467-023-39755-0)
Supplement: Supplementary file 1 — Supplementary Information [file 41467_2023_39755_MOESM1_ESM.pdf]

Supplementary Information: Quantifying Disorder One Atom at a Time Using an Interpretable Graph Neural Network Paradigm

James Chapman*[1,3], Tim Hsu†[2], Xiao Chen[2], Tae Wook Heo[1], and Brandon C. Wood‡[1]

[1]*Materials Science Division, Lawrence Livermore National Laboratory, Livermore, CA, USA*
[2]*Center for Applied Scientific Computing, Lawrence Livermore National Laboratory, Livermore, CA, USA*
[3]*Department of Mechanical Engineering, Boston University, Boston, MA*

June 16, 2023

*Corresponding Author, jc112358@bu.edu
†Corresponding Author, hsu16@llnl.gov
‡Corresponding Author, wood37@llnl.gov

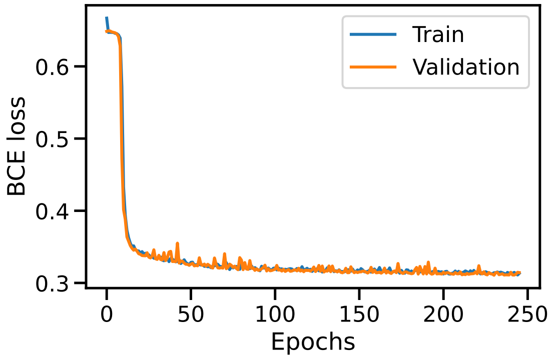

Supplementary Fig. 1: Loss curves during training. Training and validation losses are represented by the blue and orange curves, respectively. We note a convergence in both training and validation loss around 250 epochs.

Supplementary Table 1: Model parameters

Name	Notation	Value
Number of interaction layers	L	3
RBF cutoff	R_c	3.5 Å
Number of channels	D	64

Supplementary Methods

Model training

We used PyTorch Geometric [1] to develop the GNN, with the model parameters described in Table 1. The model was trained with the Adam optimizer [2], carried out using PyTorch [3] and PyTorch Geometric [1] on a NVIDIA V100 (Volta) GPU. The binary cross entropy was used as the loss function during training. The training parameters are described in Table 2. All other parameters, if unspecified in this work, default to values per PyTorch 1.8.1 and PyTorch Geometric 1.7.2.

The training dataset is based on a CMD trajectory of 1000 snapshots, from which 50 were randomly sampled for model validation. The training and validation losses are shown in Fig. 1.

Supplementary Table 2: Training parameters

Name	Notation	Value
Batch size	M	16
Number of epochs	N_{ep}	150
Learning rate	η	0.0001
First moment coefficient for Adam	β_1	0.9
Second moment coefficient for Adam	β_2	0.999

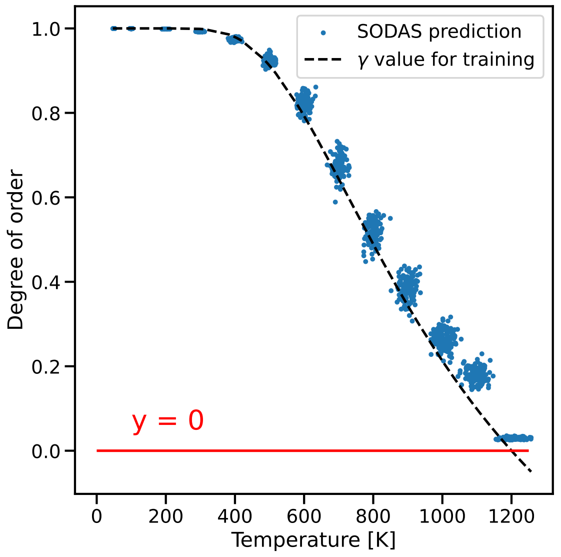

Supplementary Fig. 2: SODAS calculations on bulk structures taken during a superheating MD simulation. Values along the y-axis represent the average SODAS value for each structure in the model's training set. The dashed line indicates the theoretical values of γ while the plotted SODAS values represent the accuracy of the GNN mapping.

Model validation

Fig. 2 compares the GNN learned SODAS values on the training data with respect to the theoretical values for γ. Here, we can see excellent agreement between the GNN mapping and γ through T =1200K. This indicates that our MD simulations are sufficient to capture the configurational entropy present within the material at these temperatures.

Above T =1100K, we observe a nearly identical average SODAS value predicted between 900K and 1200K. This can be explained by by the true melting temperature of the chosen EAM potential (1050K). Therefore, above 1050K there exists a nearly identical level of local disorder compared to structures at 1200K. SODAS accurately captures this, indicating both th strength of our γ-to-λ formalism, but also the GNN's ability to accurately encode local environments in the liquid phase.

Supplementary Figures

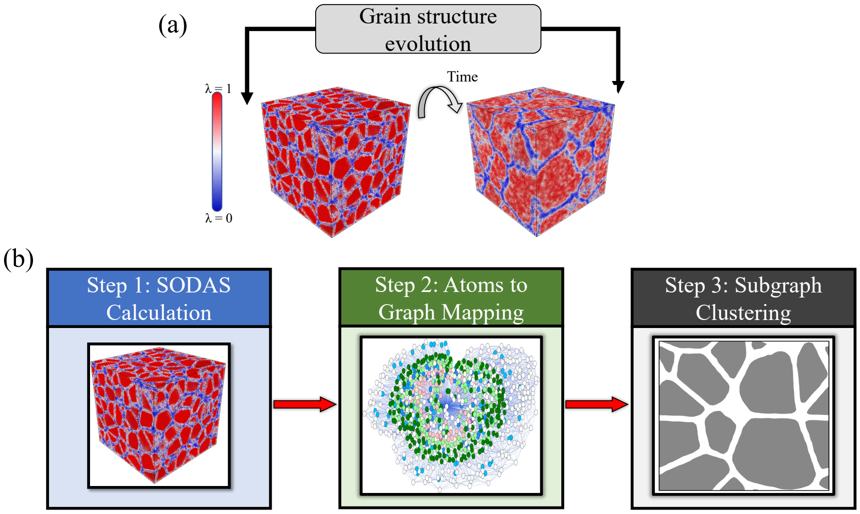

Supplementary Fig. 3: Visualization of the SODAS-to-microstructure characterization scheme. (a) SODAS predictions on the initial and final configurations of the polycrystalline MD simulation. The left image represents the initial grain structure, with the right image being the final MD snapshot. (b) Workflow of the unsupervised graph-based grain detection algorithm, visualized using simulated polycrystalline Al. (Left) SODAS values for each atomic environment present in the system. (Middle) Atoms-to-graph mapping (after SODAS thresholding), where node colors represent the connectivity of a given atom. (Right) Autonomous grain detection using recursive subgraph clustering.

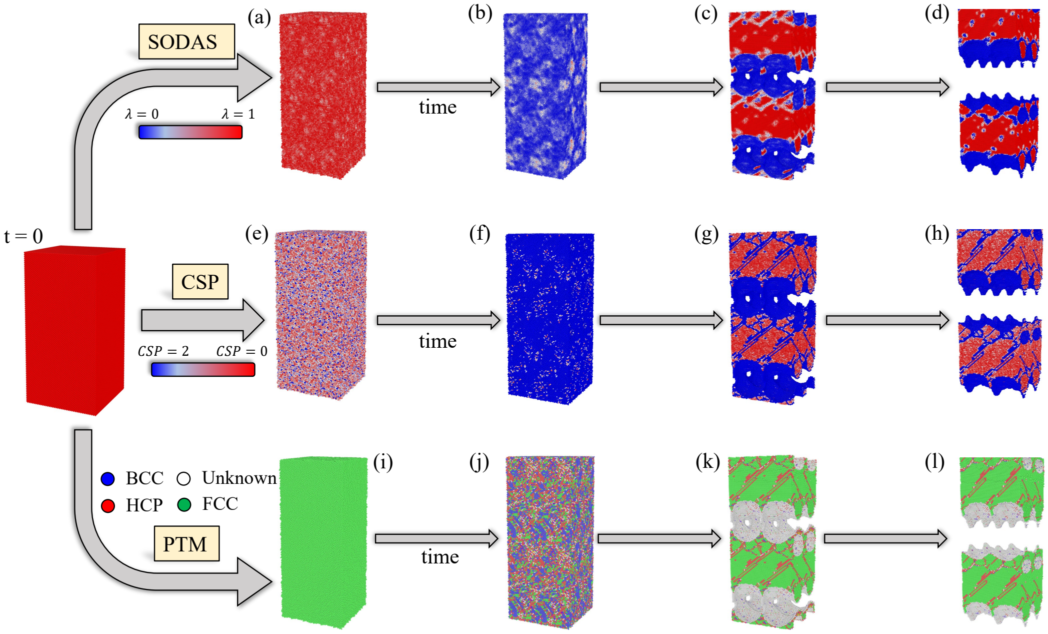

Supplementary Fig. 4: Dynamic fracture simulations shown as several snapshots throughout the process for several methods including (a-d) SODAS, (e-h) CSP, and (i-l) PTM. SODAS colorbar is shown to the left of (a), CSP colorbar shown to the left of (e), and PTM labels shown to the left of (i). Columns correspond to the same structure (ex: (a), (e), and (i) are the same structure characterized with different methods). All methods start from the same structure, shown to the left as $t = 0$.

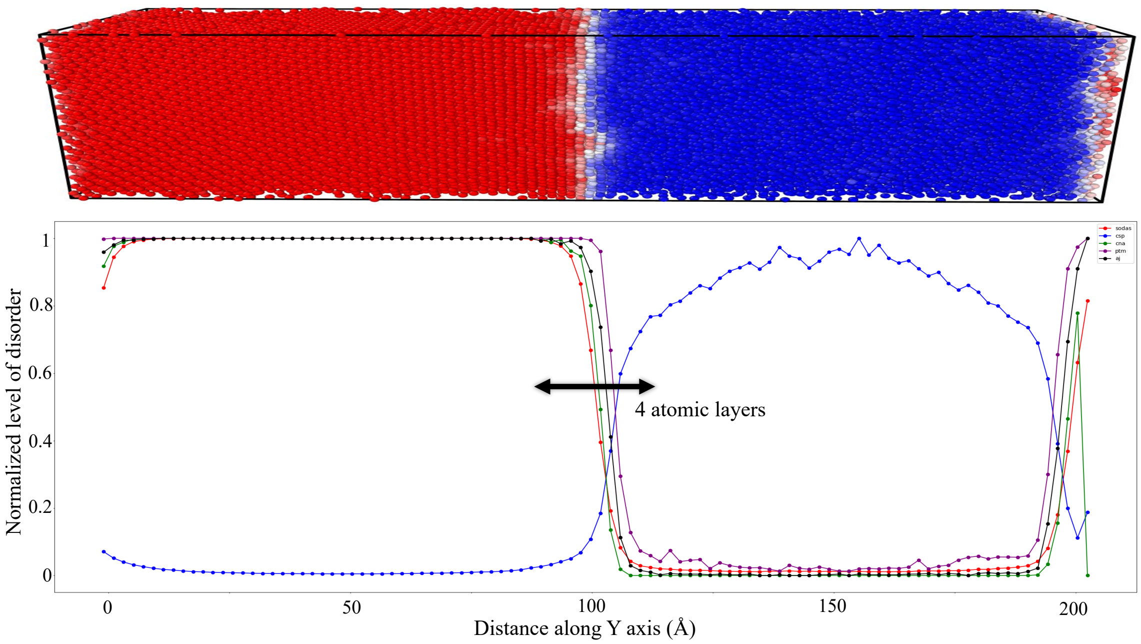

Supplementary Fig. 5: Normalized average order parameter value as a function of the distance along the y-axis. Colors correspond to a given characterization scheme. Image above represents the atomic picture of the solid liquid interface region and is color coded based on SODAS values, with red representing ordered atoms, blue representing disordered atoms, and other shades representing a point between absolute order/disorder.

Data Availability

All data required to reproduce this work can be requested by contacting the corresponding author.

Code Availability

The SODAS code can be downloaded at https://github.com/LLNL/graphite.

Acknowledgements

J. Chapman, T. Hsu, X. Chen, T. W. Heo, and B. C. Wood are partially supported by the Laboratory Directed Research and Development (LDRD) program (20-SI-004) at Lawrence Livermore National Laboratory. This work was performed under the auspices of the US Department of Energy by Lawrence Livermore National Laboratory under contract No. DE-AC52-07NA27344. J. Chapman also acknowledges the support of the Department of Mechanical Engineering at Boston University.

Author Contributions

X. Chen and B. C. Wood supervised the research. J. Chapman performed all MD simulations, and devised/implemented the autonomous microstructure feature extraction methodology. T. Hsu trained the GNN and performed all GNN-related predictions. J. Chapman and T. Hsu devised the theoretical SODAS framework. B. Wood, T. Hsu, and J. Chapman devised the atoms-to-field mapping, while T. Hsu implemented it. T. W. Heo provided insight into the connection between atomistic and phase field modelling, and helped guide discussions surrounding the atoms to continuous field methodology. J. Chapman and T. Hsu wrote the manuscript with inputs from all authors.

Supplementary References

1. Fey, M. & Lenssen, J. E. Fast graph representation learning with PyTorch Geometric. *arXiv preprint arXiv:1903.02428* (2019).
2. Kingma, D. P. & Ba, J. Adam: A method for stochastic optimization. *arXiv preprint arXiv:1412.6980* (2014).
3. Paszke, A. *et al.* Pytorch: An imperative style, high-performance deep learning library. *Advances in neural information processing systems* **32,** 8026–8037 (2019).
